# Decline in abundance and apparent survival rates of fin whales (*Balaenoptera physalus*) in the northern Gulf of St. Lawrence

**DOI:** 10.1002/ece3.5055

**Published:** 2019-03-15

**Authors:** Anna Schleimer, Christian Ramp, Julien Delarue, Alain Carpentier, Martine Bérubé, Per J. Palsbøll, Richard Sears, Philip S. Hammond

**Affiliations:** ^1^ Sea Mammal Research Unit, Scottish Oceans Institute University of St Andrews Fife UK; ^2^ Marine Evolution and Conservation, Groningen Institute for Evolutionary Life Sciences University of Groningen Groningen The Netherlands; ^3^ Mingan Island Cetacean Study St Lambert Québec Canada; ^4^ Center for Coastal Studies Provincetown Massachusetts

**Keywords:** abundance, capture heterogeneity, capture–recapture, fin whale, site fidelity, survival rate, terminal bias

## Abstract

Estimates of abundance and survivorship provide quantifiable measures to monitor populations and to define and understand their conservation status. This study investigated changes in abundance and survival rates of fin whales (*Balaenoptera physalus*) in the northern Gulf of St. Lawrence in the context of anthropogenic pressures and changing environmental conditions. A long‐term data set, consisting of 35 years of photo‐identification surveys and comprising more than 5,000 identifications of 507 individuals, formed the basis of this mark–recapture study. Based on model selection using corrected Akaike Information Criterion, the most parsimonious Cormack–Jolly–Seber model included a linear temporal trend in noncalf apparent survival rates with a sharp decline in the last 5 years of the study and a median survival rate of 0.946 (95% confidence interval (CI) 0.910–0.967). To account for capture heterogeneity due to divergent patterns of site fidelity, agglomerative hierarchical cluster analysis was employed to categorize individuals based on their annual and survey site fidelity indices. However, the negative trend in survivorship remained and was corroborated by a significant decline in the estimated super‐population size from 335 (95% CI 321–348) individuals in 2004–2010 to 291 (95% CI 270–312) individuals in 2010–2016. Concurrently, a negative trend was estimated in recruitment to the population, supported by a sharp decrease in the number of observed calves. Ship strikes and changes in prey availability are potential drivers of the observed decline in fin whale abundance. The combination of clustering methods with mark–recapture represents a flexible way to investigate the effects of site fidelity on demographic variables and is broadly applicable to other individual‐based studies.

## 1 INTRODUCTION

Detecting trends in population abundance and identifying the underlying factors driving any increase or decline in population size are important aspects of conservation biology and wildlife management (Lawton, 1993; Taylor & Gerrodette, 1993). Small populations that occur at low population densities and/or occupy restricted geographical ranges face an enhanced extinction risk and are especially in need of focussed monitoring (Purvis, Gittleman, Cowlishaw, & Mace, 2000). In this context, abundance and survival rate estimates provide quantifiable measures to define the status of populations and to assess the efficiency of management actions (Cheney et al., 2014; Pace, Corkeron, & Kraus, 2017). While a time series of abundance estimates can reveal population trends, reproductive and survival rates can provide insights into causes of observed changes in population abundance (Pace et al., 2017; Pendleton et al., 2006; Ramp, Delarue, Bérubé, Hammond, & Sears, 2014). In marine vertebrates, robust estimation of reproductive (birth) rates is available for several seabird, sea turtle and pinniped species, facilitated by the confinement to terrestrial birthing and breeding colonies (Cury et al., 2011; Pomeroy, Fedak, Rothery, & Anderson, 1999; Troëng & Rankin, 2005). In cetaceans, robust estimates of birth rates are more difficult to obtain and are limited to a few well studied populations (e.g., Arso Civil, Cheney, Quick, Thompson, & Hammond, 2017; Mann, Connor, Barre, & Heithaus, 2000; Rolland et al., 2016).

Well‐established analytical frameworks are available to generate robust estimates of abundance and survival from suitable data to inform decision‐making (Hammond, 2017; Thomas et al., 2010). However, the statistical power to detect population trends depends, among other factors, on the life history of the species under investigation (Taylor & Gerrodette, 1993; Thompson, Wilson, Grellier, & Hammond, 2000). In particular, population assessments in long‐lived and wide‐ranging species pose logistical challenges relating to monitoring regimes and spatial coverage (Taylor, Martinez, Gerrodette, Barlow, & Hrovat, 2007; Tyne et al., 2016). First, long‐term monitoring programmes increase the power to detect trends in long‐lived species. For instance, Wilson, Hammond, and Thompson (1999) calculated that research effort during more than 8 years was required to detect changes in bottlenose dolphin population abundance with a statistical power at 90%. Second, as a result of limited resources, surveys often cover a small part of a population's distribution, although exceptions include large‐scale surveys covering the North Atlantic (Lockyer & Pike, 2009), Antarctic waters (e.g., Branch, 2007), and the eastern tropical Pacific (Gerrodette & Forcada, 2005). In studies with restricted spatial coverage, it is difficult to interpret changes in estimated abundance as true changes in population size because of natural shifts in distribution or because of temporary emigration from the study area (Cheney et al., 2013; Forney, 2000; Víkingsson et al., 2015; Wilson, Reid, Grellier, Thompson, & Hammond, 2004).

The fin whale (*Balaenoptera physalus*) is a long‐lived and wide‐ranging baleen whale requiring large‐scale, long‐term monitoring programmes to identify population trends. Several national and multinational large‐scale surveys (e.g., NASS (Lockyer & Pike, 2009), SCANS (Hammond et al., 2017), NAISS (NAMMCO, 2018)) have focussed on assessing long‐term changes in distribution and abundance of cetaceans, including fin whales, in the North Atlantic. For instance, in comparison with previous estimates, a substantial increase in fin whale abundance was reported in the Irminger Sea (between Iceland and Greenland), possibly as a result of shifts in distribution and prey availability (Víkingsson et al., 2015). The sum of available abundance estimates from the most recent large‐scale surveys adds up to more than 70,000 fin whales in the North Atlantic (NAMMCO, 2018). While fin whales are known to range over the whole North Atlantic basin, they also show strong site fidelity to specific feeding grounds (Agler, Schooley, Frohock, Katona, & Seipt, 1993; Ramp et al., 2014). Long‐term small‐scale studies may therefore hold valuable information on changes in local abundances and allow for more detailed investigation into recruitment and survival rates.

A study of fin whales in the northern part of the Gulf of St. Lawrence (GSL) during 2004 to 2010 that applied mark–recapture models to photo‐identification data estimated the population at 328 individuals (with 95% confidence interval (CI) of 306–350; Ramp et al., 2014). This study found that noncalf apparent survival probabilities remained stable at 0.955 (95% CI 0.936–0.969) during the period from 1990 to 2006 but there were indications of a decrease during 2007 to 2010. A combination of lower site fidelity and elevated mortality rates were discussed as potential causes of declining survivorship. Mark–recapture models assume homogeneity in capture probabilities at a given sampling occasion, unless individual variation is explicitly modeled (Lebreton, Burnham, Clobert, & Anderson, 1992). This assumption is frequently violated in practice in studies of cetaceans because capture probabilities can vary among individuals as a function of intrinsic factors associated with biological or behavioral characteristics (e.g., age, sex, or site fidelity) and of extrinsic factors such as the sampling design (Hammond, 2017). For instance, sampling only a fraction of the distribution when individuals display site fidelity can lead to unequal capture probabilities. Such capture heterogeneity can bias results, typically leading to an underestimation of abundance (Hammond, 1990a). It is unclear to what extent previous estimates of fin whale survival probabilities and abundance were biased due to capture heterogeneity.

The level of connectivity between fin whales in the GSL and neighboring areas is unresolved. Whaling reports (Sergeant, 1977), contaminant levels (Hobbs, Muir, & Mitchell, 2001), and song structure (Delarue, Todd, Parijs, & Iorio, 2009) suggest that the GSL individuals form a distinct population. However, photo‐identification and genetic studies do not fully support this hypothesis, instead pointing to exchange with surrounding areas (Bérubé et al., 1998; Coakes et al., 2005). While population identity remains unresolved, several studies suggested that the GSL has been undergoing a substantial change with wide‐ranging effects on its fauna (Friesinger & Bernatchez, 2010; Plourde et al., 2014). A shift in the arrival date of fin and humpback (*Megaptera novaeangliae*) whales to their feeding ground in the GSL has been related to earlier winter sea ice break‐up linked to a warming climate (Ramp, Delarue, Palsbøll, Sears, & Hammond, 2015). Significant changes in the ichthyoplankton community structure (Bui, Ouellet, Castonguay, & Brêthes, 2010), unprecedented warming of the incoming intermediate North Atlantic water (Thibodeau, Vernal, Hillaire‐Marcel, & Mucci, 2010), and higher mortality in harp seals (*Pagophilus groenlandicus*) linked to reduced ice cover (Johnston, Bowers, Friedlaender, & Lavigne, 2012) are also indicative of ecosystem changes.

The aforementioned lack of recent abundance estimates, the possible decline in survival rates, and ongoing environmental and anthropogenic pressures in the GSL highlight the need for updated estimates of abundance and survival in fin whales. These estimates are crucial to detect local population changes in the GSL, which may otherwise remain undetected in the framework of large‐scale surveys. This study aimed to fill this gap in population trends of fin whales in the GSL, providing key information for future assessments of the population status in Atlantic Canadian waters. A long‐term data set, consisting of 35 consecutive years of photo‐identification surveys in the northern GSL, formed the basis of this study. Our specific objective was to test the hypothesis that there is an ongoing decline in numbers and survival of fin whales in the GSL. Results are discussed in the context of anthropogenic pressures and environmental changes.

The impact of capture heterogeneity on estimates of population parameters is not unique to cetacean individual‐based studies and has long been recognized as a potential source of bias in various taxa, for example, seabirds (Sanz‐Aguilar et al., 2010) and pinnipeds (Bradshaw, Barker, Harcourt, & Davis, 2003). While many studies acknowledge the problem, few studies have assessed the magnitude of bias introduced by capture heterogeneity. The analysis presented here focussed on reducing the effect of possible sources of bias resulting from capture heterogeneity and temporary emigration to provide robust results. The proposed method used to categorize individuals based on site fidelity indices has the potential for broad applicability to other individual‐based studies.

## 2 MATERIAL AND METHODS

### 2.1 Study area and field data collection

**Figure 1:**
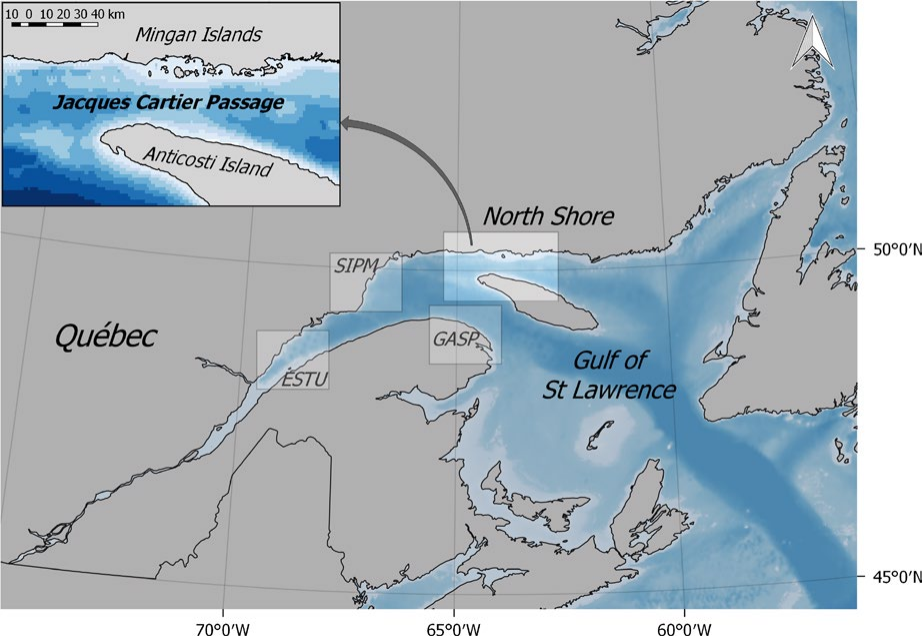
Location of the study area in the Jacques Cartier Passage, situated between Anticosti Island and the North Shore on the East coast of Canada. Opportunistic photo‐identification data were available for Gaspé (GASP), the St. Lawrence Estuary (ESTU), and Sept‐Iles to Pointe‐des‐Monts (SIPM)

The study area of approximately 8,000 km^2^ was situated in the Jacques Cartier Passage (JCP), located between Anticosti Island and the North Shore in the northern part of the GSL (Figure 1). The area is characterized by upwelling and high productivity, forming a feeding ground for several baleen whale species (Doniol‐Valcroze, Berteaux, Larouche, & Sears, 2007; El‐Sabh, 1976). Since 1982, the Mingan Island Cetacean Study has conducted annual surveys in the area to collect photo‐identification data from fin whales. Data collection was conducted from late May/early June until late September/October, with an average of 50 survey days and 13,000 km per year. Surveys were conducted from 7 m long rigid‐hulled inflatable boats and aimed to cover a large area, while maximizing encounter rates for photo‐identification by spending more time in areas with high numbers of individuals. Realized survey effort depended on weather conditions and was discontinued when sea conditions were worse than Beaufort scale 3 or visibility was less than one nautical mile. During the period from 1982 to 2003, photographs were obtained using standard single lens reflex (SLR) 35 mm cameras with black and white film, and from 2004 onwards with digital SLR cameras, with 70–200 mm lenses. From 1990 onwards, skin biopsy samples were collected from free‐ranging individuals (Palsbøll, Larsen, & Sigurd Hansen, 1991) for molecular determination of sex from the DNA extracted from skin samples using the ZFX/ZFY chromosomes following the methodology described in Bérubé and Palsbøll (1996a, 1996b).

### 2.2 Photo‐identification data

**Figure 2:**
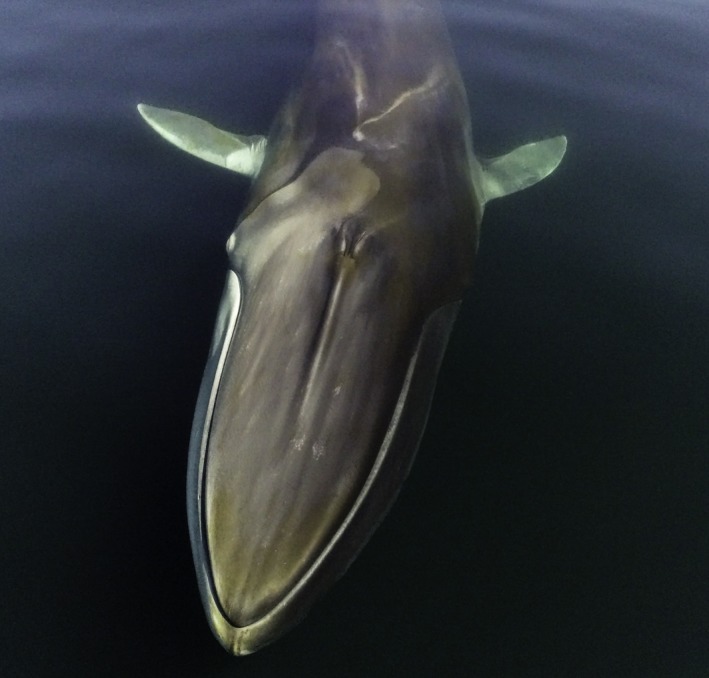
Overhead view of a fin whale (*Balaenoptera physalus*) in the Gulf of St. Lawrence. The typical asymmetrical pigmentation was used for photo‐identification. Photograph credit: TerreSky/MICS

Photo‐identification (photo‐ID) is a method in which individual animals are recognized from images of their natural markings, providing capture histories of individuals (Hammond, 1990b). Photo‐ID methodology has been successfully used in previous studies of fin whales (Agler et al., 1990, 1993; Ramp et al., 2014; Robbins et al., 2007; Whooley, Berrow, & Barnes, 2011). Individual fin whales are identified based on the profile of the dorsal fin and the unique pigmentation pattern of the so‐called chevron (Agler et al., 1990). The chevron is a light “V”‐shaped pigmentation pattern, which extends from the blow holes down both sides of the animal, but is more pronounced on the right side (Figure 2).

In this study, every fin whale was considered individually identifiable from high‐quality photographs of the shape of the dorsal fin and the pigmentation patterns. After each survey, the best photos of the right side dorsal and right side chevron were chosen for each individual based on sharpness, angle, coverage, and light conditions (contrast). Based on photo quality, pictures were graded from A to D and only individuals with pictures from the highest quality (A and B) categories were matched against the catalogue. In an initial step, researchers matched individuals to individuals already seen during the same season to check for internal duplicates, before matching the individuals seen in a season to the whole catalogue of previously identified individuals. The judgment of three researchers, two of whom had to be experienced in fin whale matching, was required to confirm a match or designate a new individual in order to minimize the chance of mis‐identification of individuals. Calves were excluded from mark–recapture analyses because observations of calves are conditional on the presence of their mother.

### 2.3 Residency in the study area

The complete sighting history for each individual was used to calculate two site fidelity indices: (a) annual sighting rates as the total number of years an individual was seen as a proportion of the total number of years since the first sighting of the given individual and (b) survey sighting rates as the total number of days an individual was seen as a proportion of the total number of survey days carried out since the first sighting of the given individual. Thus, an annual sighting rate of 1 indicated that an individual had at least one confirmed identification each year since the first sighting. The survey sighting rates were related to seasonal residency patterns; individuals identified multiple times in a single season scored higher than individuals sighted only once. Individuals first identified after 2011 were excluded from the analysis because insufficient time had passed to estimate a robust site fidelity index.

Agglomerative hierarchical cluster (AHC) analysis was used to categorize individuals based on the two site fidelity indices using the *hclust* function in R (version 3.4.3, R Core Team, 2015). AHC analysis was chosen over alternative clustering and ordination methods because it follows a bottom‐up approach that does not require prior specification of the expected number of clusters. The method has been applied to study residency patterns in other marine vertebrates (Daly, Smale, Cowley, & Froneman, 2014; Zanardo, Parra, & Möller, 2016). The site fidelity indices were standardized relative to the median and the median absolute deviation using the *scale* function in R, in order to enable comparisons of the two indices. The AHC analysis requires specification of an agglomerative clustering algorithm and measure of dissimilarity. Here, Ward's method was applied as the clustering algorithm because of its known robustness and competitiveness with other clustering methods (Cao, Bark, & Williams, 1997; Murtagh & Legendre, 2014; Ward, 1963). Ward's method was formulated based on Euclidean distance, which was applied as a measure of dissimilarity to create a distance matrix. The AHC analysis compares all distances between all the observations and new clusters are formed by pairing the closest observations. This process is repeated until a single cluster remains. The cluster solution was displayed as a dendrogram, which was inspected visually to choose the number of site fidelity groups (see below).

As a complement to the above analyses, confirmed opportunistic matches of fin whales between the JCP and adjacent areas were considered as a means to improve the understanding of the ranging capacities of the animals within and among seasons.

### 2.4 Mark–recapture analyses

Cormack–Jolly–Seber (CJS; Pollock, Nichols, Brownie, & Hines, 1990) models were fitted to estimate apparent survival probabilities (Φ) and recapture probabilities (*p*), and the POPAN formulation for the Jolly–Seber model (Schwarz & Arnason, 1996) was employed to estimate the abundance of the super‐population *N*. Here, the super‐population is defined as the total number of individuals that were in the study area at some point from 2010 to 2016. CJS and POPAN models were fitted using the R package RMark (version 2.2.2, Laake & Rexstad, 2008). Each field season was considered a single sampling occasion, resulting in a total of 35 capture–recapture occasions from 1982 to 2016.

Goodness‐of‐fit (GOF) tests implemented in the software U‐CARE (version 2.3.2, Choquet, Lebreton, Gimenez, & Pradel, 2005) were applied to assess the fit of a fully parameterised CJS model to the data and to identify unequal survival or recapture probabilities, for example as a result of transients (Pradel, Hines, Lebreton, & Nichols, 1997) or trap‐dependence (trap‐happiness or trap‐shyness; Pradel, 1993). Transients were defined as individuals with a single sighting, thus having zero probability of survival after their initial capture (Pradel et al., 1997). Over‐dispersion in the data was estimated as the ratio, $\hat{c}$, of the overall Pearson χ^2^ statistic to its number of degrees of freedom.

Following the methodology described by Ramp et al. (2014), CJS models were fitted to estimate apparent survival and recapture probabilities of sexed animals from 1990 to 2015. Information on the sex of individuals was limited to a fraction of the data set from 1990 to 2015. To account for potential bias introduced by limiting the dataset to biopsied individuals, individuals were assumed to enter the population during the year they were biopsied and all previous sightings were discarded (Nichols, Kendall, Hines, & Spendelow, 2004). The probability of apparent survival Φ*_t_* is the product of two components: (a) the probability that an individual survives from occasion *t* to *t* + 1 and (b) the probability that the individual returns to the study area (site fidelity). Candidate models were built based on different combinations of effects on survival and recapture probabilities following the notation of Lebreton et al. (1992): constant over sampling occasions (·), fully time‐dependent (*t*), linear temporal trend (*T*), sex (*s*), and trap‐dependence (*m*). The inclusion of immediate trap‐dependence in the models of recapture probabilities was justified by the lack of fit in the Test2.CT component of the GOF test (U‐CARE Test2.CT, χ^2^ = 53.40, *df* = 23, *p* < 0.001). The effect of trap‐dependence was incorporated as an individual time‐varying covariate using dummy variables (0 and 1), which allowed capture probabilities to vary depending on whether the individual was captured on the previous occasion or not (Huggins, 1989). No transient effect (U‐CARE Test3.SR, χ^2^ = 8.92, *df* = 17, *p* = 0.94) or over‐dispersion (overall χ^2^ = 154.51, *df* = 152; $\hat{c}$ = 1.01) was detected in the data. In addition to the strictly linear temporal trend, a more flexible temporal trend with multiple inflection points (knots) was explored using the *bs()* function in the R *splines* package (Altukhov et al., 2015). The optimal number of knots was chosen by comparing models with one to six knots based on the Akaike Information Criterion corrected for small sample size (AIC_C_, Anderson, Burnham, & White, 1994). The model with three knots was retained. Additive (+) and interaction (:) effects were also considered in the form of *p_t_*_ +_ *_m_* and Φ*_s_*_:_ *_T_*.

Two potential sources of bias in survival estimation were assessed: temporary emigration and capture heterogeneity. As long as temporary emigration is random, survival estimates in CJS models remain unbiased (Schaub, Gimenez, Schmidt, & Pradel, 2004); however, problems arise when nonrandom (Markovian) temporary emigration occurs, which can introduce severe bias, particularly at the end of the time series (terminal bias: Kendall, Nichols, Hines, & Mar, 1997; Langtimm, 2009). Especially in the case of long‐lived, highly mobile species, Markovian temporary emigration from the study area may not be uncommon (Langtimm, 2009). As a result, the increased uncertainty about the fate (death or temporarily unavailable for capture) of individuals at the end of the time series can make the interpretation of temporal trends in survival probabilities difficult (Peñaloza, Kendall, & Langtimm, 2014). As suggested by Langtimm (2009), we truncated the original capture histories and reanalyzed the data over shorter time periods in order to assess whether the original data set was subject to terminal bias.

The assumption of equal capture probabilities may be violated due to differences in site fidelity patterns. To investigate the effect of heterogeneous site fidelity patterns, we estimated apparent survival probabilities for the different site fidelity clusters (i.e., core regulars and occasional visitors (see below)) as identified by the AHC analysis. CJS models were fitted to the categorized capture histories from all noncalf individuals (sexed and unsexed) from 1990 to 2016. As was the case for the AHC analysis, individuals that entered the data set during the last 5 years of the study were excluded.

Because there was little support for sex‐specific survival rates (see below), sighting histories from all noncalf individuals (sexed and unsexed) identified during 2010 to 2016 were used to provide an abundance estimate of the super‐population *N* using POPAN. In addition to the apparent survival Φ and recapture *p *probability parameters, the POPAN formulation also estimates the probability of entry (*pent*) from the super‐population into the study area, as a result of immigration and/or birth. We hereafter refer to this as recruitment. The parameters Φ, *p*, and *pent* were allowed to be constant (·), time‐dependent (*t*), or to follow a linear temporal trend (*T*). A CJS model was used as an approximation for GOF testing, because U‐CARE does not support GOF tests of POPAN models. While there was no evidence for an effect of trap‐dependence (U‐CARE Test2.CT; χ^2^ = 4.87, *df* = 4, *p* = 0.301), the GOF tests indicated the presence of transients in the dataset (U‐CARE Test3.SR; χ^2^ = 23.80, *df* = 5, *p* < 0.001). To account for a potential transient effect due to the inclusion of all individuals, we fitted models with separate survival for the interval following the first sighting of a whale by creating a time‐varying covariate called *trans* (cf. Laake & Rexstad, 2008, Félix, Castro, Laake, Haase, & Scheidat, 2011). For each individual, seven (one for each year) covariates were created with 1 for an animal's first sighting and 0 for all other occasions. After accounting for transience, the over‐dispersion factor was estimated to be $\hat{c}$ = 1.4 and the model selection criterion (QAIC_C_) and estimated standard errors were adjusted accordingly. Additive (+) and interaction (:) effects were also considered for the survival model (Φ_*trans* +_ *_T_* and Φ_*trans*:_ *_T_*). Because the estimate previously published by Ramp et al. (2014) did not include a transient effect, the POPAN models with transient effect were rerun with the 2004 to 2010 data to provide a reference estimate for comparison.

Model selection of candidate CJS and POPAN models was based on AIC_C_ and Quasi‐likelihood AIC_C_ (QAIC_C_, Burnham & Anderson, 2002), respectively. Model‐averaged estimates were computed based on the AIC_C _or QAIC_C_ weight of the models.

## 3 RESULTS

**Figure 3:**
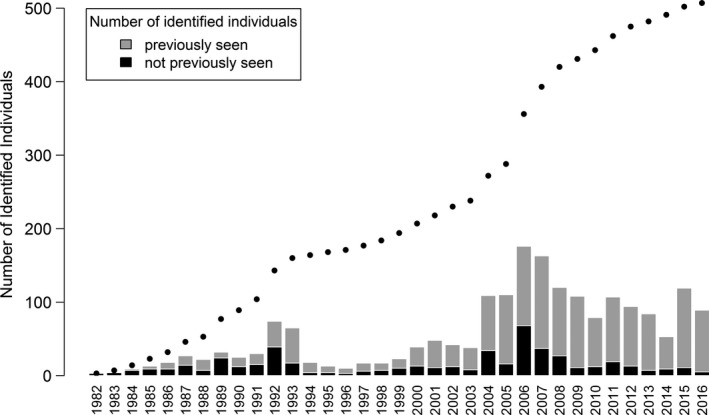
Discovery curve of the cumulative number of identified individuals in the catalogue from 1982 to 2016 (points), with bar plots showing the number of previously identified and new individuals seen each year

In total, 5,191 identifications of 507 individual fin whales were made from 1982 to 2016. The number of identified individuals in the catalogue increased steadily since 1982, marked by increased effort during 1992 and 1993, as well as the introduction of digital SLR cameras in 2004 (Figure 3). After a pronounced increase in new identifications during 2004 to 2008, the rate of new identifications decreased after 2008. Sex was determined for 196 individuals, of which 74 were females and 122 males. Since 2004, a total of 78 calves was reported, but the number decreased sharply after 2008 to almost none since 2013 (Sullivan‐Lord et al. in prep). Forty‐one individuals, categorized as calves on their first sighting, were excluded from mark–recapture analyses.

### 3.1 Cluster analysis

**Figure 4:**
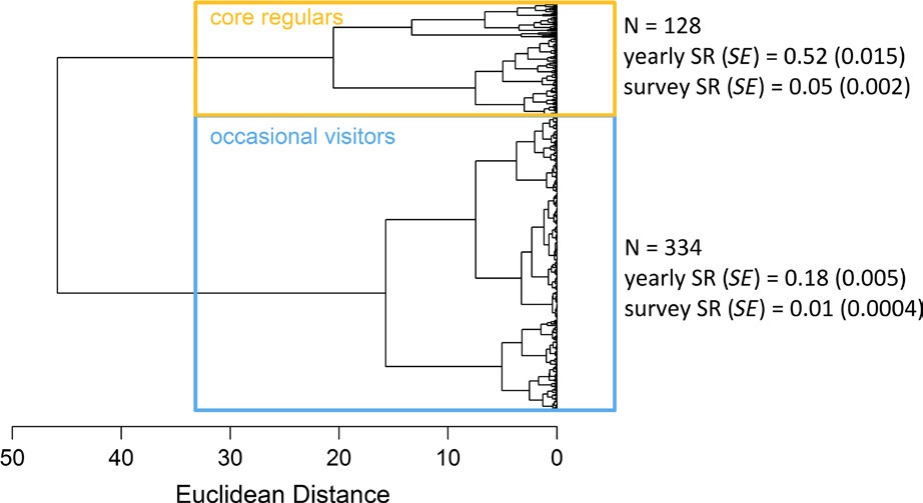
Dendrogram visualizing results of agglomerative hierarchical cluster analysis, where every tip of a branch represents one individual. Individuals can be split in two categories: a high site fidelity group (core regulars; yellow) and a low site fidelity group (occasional visitors; blue). The sample size (*n*), mean yearly and survey sighting rates (SR) with standard errors (*SE*) are given for each group

The AHC analysis separated individuals into two distinct categories (Figure 4). Out of the 462 individuals, the sighting histories of 128 individuals were characterized by high yearly and survey sighting rates, forming the core of individual fin whales that frequent the study area regularly, hereafter referred to as core regulars. The second group comprised the remaining 334 individuals, which showed very low site fidelity indices and are hereafter referred to as occasional visitors. This group included transient individuals that were only seen once, as well as individuals with a sparse re‐sighting history in the study area. This clustering solution was confirmed by significant differences in mean yearly (two‐sample *t*_159.15_ = −21.50, *p* < 0.001) and survey (two‐sample *t*_138.88_ = −19.63, *p* < 0.001) sighting rates between the two categories. The core regulars comprised 31 females and 63 males, compared to 37 females and 58 males among the occasional visitors. These sex ratios did not differ significantly between the two groups (2‐sample test for equality of proportions; χ^2^ = 0.49, *df* = 1, *p* = 0.48).

### 3.2 Exchange rates with adjacent areas

Limited photo‐identification data from outside the JCP were available to gain an insight into the movement patterns of fin whales in the GSL (Table 1). Despite restricted survey effort in the Estuary (ESTU), Gaspé (GASP), and Sept‐Iles et Pointe‐des‐Monts (SIPM), 24.5% (*n* = 129) of the individuals were identified in at least two areas in the GSL. Fifty‐one individuals were recorded in multiple areas in the same year.

**Table 1:** Number of individuals identified within a single area or across multiple areas

Area	Number of individuals identified
Matched individuals within areas	
ESTU	2
GASP	13
JCP	381
SIPM	2
Total	398
Matched individuals between areas	
ESTU + GASP	1
ESTU + JCP	26
ESTU + SIPM	2
GASP + JCP	43
JCP + SIPM	28
ESTU + GASP + JCP	14
ESTU + GASP + SIPM	1
ESTU + JCP + SIPM	5
GASP + JCP + SIPM	7
ESTU + GASP + JCP + SIPM	2
Total	129

Note

Location of additional areas shown in Figure 1. In JCP identifications were made on 821 survey days, compared to opportunistic effort of 91 survey days in GASP, 32 in SIPM, and unknown number of surveys in ESTU.

ESTU: St. Lawrence Estuary; GASP: Gaspé; JCP: Mingan Islands and Jacques Cartier Passage; SIPM: Sept‐Iles to Pointe‐des‐Monts.

### 3.3 Estimated apparent survival rates during 1990 to 2015

**Figure 5:**
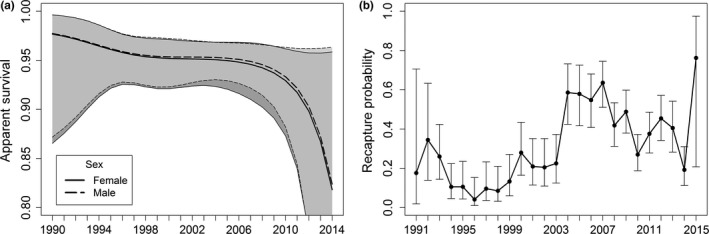
Model‐averaged (a) apparent survival Φ and (b) recapture probabilities *p* with 95% confidence intervals from the Cormack–Jolly–Seber models listed in Table 2

Model support (weight) was limited to four candidate models (Table 2: models 1–4), all of which accounted for time and trap‐dependence in the recapture probabilities; models without the interaction term of *p_t_*_ +_ *_m_* (models 5–12) had no support. The two models for survival based on temporal trends (models 1 and 3) had more support than the equivalent model without a trend (models 2 and 4). Survival models without sex (models 1 and 2) carried more weight than those with sex (models 3 and 4). Model‐averaged estimates of apparent survival and recapture probabilities are shown in Figure 5. A clear negative trend in estimated apparent survival was apparent since the start of the study (Figure 5a). An initial decline was followed by a period of little change between 1998 and 2008, followed by a sharp decline. The median overall estimate of survivorship was 0.946 (95% CI 0.910–0.967). There was little difference in estimates of survival between sexes. Recapture probabilities varied considerably among years (Figure 5b). Elevated probabilities were observed from 2004 and onwards, coinciding with the transition to digital photography.

**Table 2:** Cormack–Jolly–Seber models fitted to data from 1990 to 2015 for estimation of survival rate Φ

Model	npar	AIC_C_	ΔAIC_C_	weight	Residual deviance
1. Φ_(_ *_T_*_)_ *p*_(_ *_t_*_ + _ *_m_*_)_	30	2,015.80	0.00	0.46	1,953.04
2. Φ_(·)_ *p*_(_ *_t_*_ + _ *_m_*_)_	27	2,017.22	1.43	0.23	1,960.99
3. Φ_(s:_ *_T_*_)_ *p*_(_ *_t_*_ + _ *_m_*_)_	29	2,017.23	1.43	0.23	1,956.65
4. Φ_(_ *_s_*_)_ *p*_(_ *_t_*_ + _ *_m_*_)_	28	2,019.15	3.36	0.09	1,960.76
5. Φ_(_ *_T_*_)_ *p*_(_ *_t_*_)_	29	2,049.99	34.20	0.00	1,409.62
6. Φ_(s:_ *_T_*_)_ *p*_(_ *_t_*_)_	28	2,052.48	36.68	0.00	1,414.28
7. Φ_(_ *_·_*_)_ *p*_(_ *_t_*_)_	26	2,058.85	43.05	0.00	1,424.98
8. Φ_(_ *_s_*_)_ *p*_(_ *_t_*_)_	27	2,060.62	44.82	0.00	1,424.59
9. Φ_(_ *_·_*_)_ *p*_(_ *_m_*_)_	3	2,136.46	120.66	0.00	2,130.43
10. Φ_(_ *_s_*_)_ *p*_(_ *_m_*_)_	4	2,138.43	122.63	0.00	2,130.37
11. Φ_(s:_ *_T_*_)_ *p*_(_ *_m_*_)_	5	2,139.99	124.19	0.00	2,129.90
12. Φ_(_ *_T_*_)_ *p*_(_ *_t_*_)_	6	2,141.45	125.66	0.00	2,129.33

Note

Models are ordered in ascending order of their Akaike Information Criterion corrected for small sample sizes (AIC_C_); npar: number of parameters; ΔAIC_C_: difference in AIC_C_ in relation to model with lowest AIC_C_; *p*: recapture probability; *T*:linear temporal trend; *t*: time (sampling occasion); *m*: trap‐dependence; *s*: sex; (·): constant.

**Figure 6:**
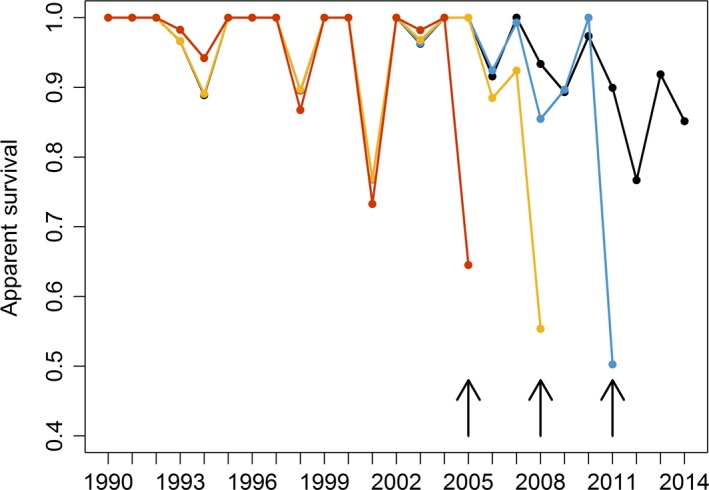
Temporal trend in survival probabilities of the full 25‐year period (black) and of capture histories truncated to 22 (blue), 19 (yellow), and 16 (red) years. Arrows indicate the last survival value of truncated time series. The truncation of capture histories was used to investigate the presence of terminal bias in survival probabilities. Based on time‐dependent CJS model Φ*_t_p_t_*_ +_ *_m_*

Truncation of the data set to shorter time periods strongly suggested a terminal bias due to nonrandom temporary emigration (Langtimm, 2009; Figure 6). When sighting histories were truncated by 3, 6, and 9 years, the apparent survival estimates of the last 1 to 3 years were lower than the estimates for the same period with the full data set. The large proportion of occasional visitors identified during the AHC analysis might be the cause of the terminal bias.

**Figure 7:**
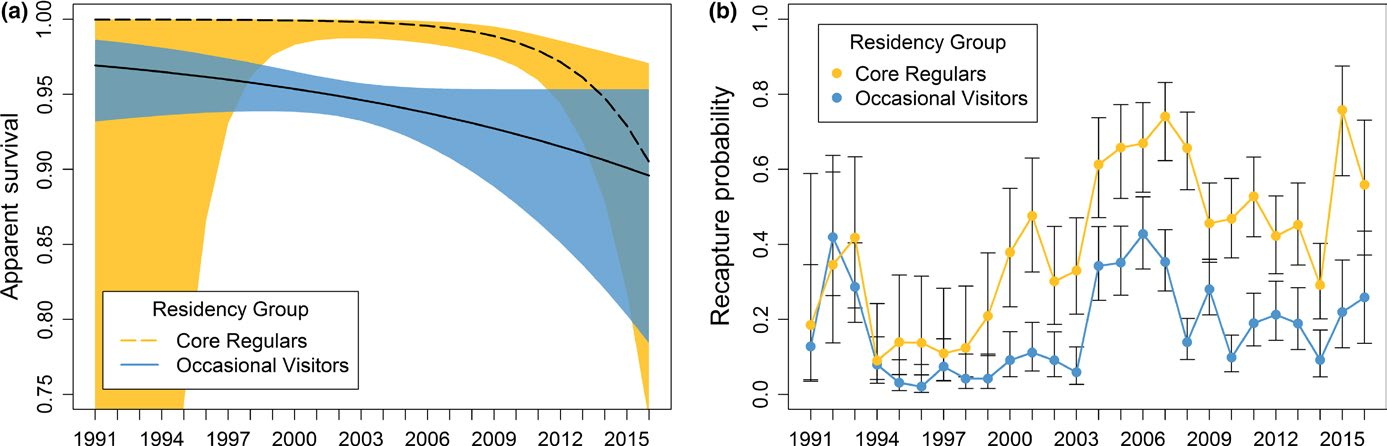
Time series of model‐averaged (a) apparent survival Φ and (b) recapture probabilities *p* with 95% confidence intervals. Cormack–Jolly–Seber models (Supporting Information Table A1 + A2) were fitted separately to sighting histories of core regulars and occasional visitors

To test the hypothesis of terminal bias caused by divergent site fidelity patterns, CJS models were fitted separately to the sighting histories of the core regulars and the occasional visitors (Supporting Information Table A1 + A2). As expected, the recapture probabilities of the high site fidelity core regulars were significantly higher than those of the low site fidelity occasional visitors (Figure 7b), indicating that the assumption of recapture homogeneity was violated in the previous model where all individuals were pooled together (see Figure 5b). Apparent survival probabilities were overall lower for the occasional visitors and showed a constant negative trend (Figure 7a). For the highly site faithful core regulars, apparent survival probabilities were close to one before declining from 2005 and onwards.

### 3.4 Estimated super‐population size

**Figure 8:**
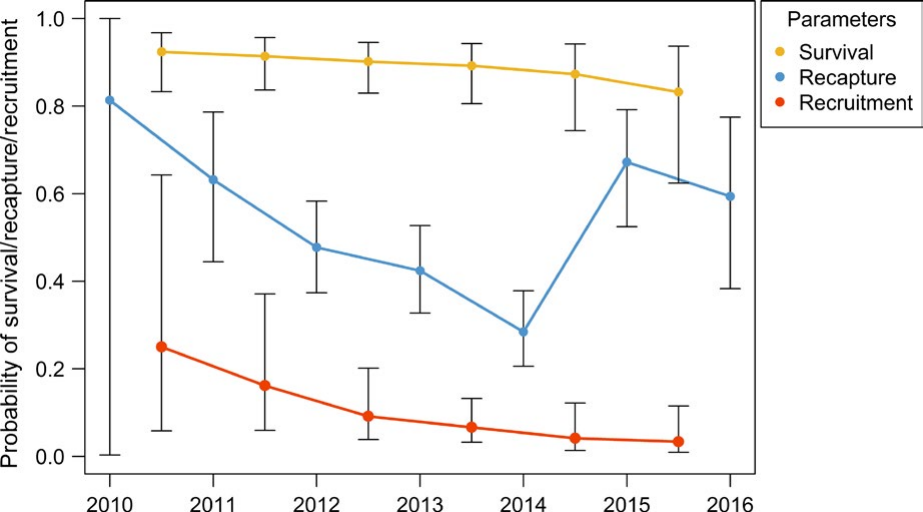
Model‐averaged probabilities of apparent survival Φ (yellow), capture *p* (blue), and recruitment *pent* (red) with 95% confidence intervals for the POPAN models from 2010 to 2016 (Table 3)

The most supported model included the transient effect and temporal trend for the probability of survival Φ, with time‐dependent recapture probability *p* and a linear temporal trend for recruitment *pent* (Table 3 model 1). All candidate models which included the transient effect in the survival model (models 1–9) had greater support (based on QAIC_C_ weight) than comparable models without the transient effect (models 10–12), highlighting the importance of this additional parameter. The probabilities of apparent survival and recruitment steadily decreased over time, with recruitment almost reaching zero probability by the end of the time series (Figure 8). Recapture probabilities reached a minimum of 0.3 in 2014 and increased to ~0.6 during the last 2 years of the study. The model‐averaged estimate of super‐population size *N *was 291 (95% CI 270–312), reflecting the estimate of abundance of the population which contributed individuals to the study area during 2010 to 2016. The same models fitted to the 2004 to 2010 data resulted in a model‐averaged super‐population size of 335 (95% CI 321–348) from 2004 to 2010 (Supporting Information Table A3).

**Table 3:** Selection of POPAN models fitted to data from 2010 to 2016 for estimation of super‐population size *N*

Model	npar	QAIC_C_	ΔQAIC_C_	Weight	QDeviance
1.$\Phi_{\left(\mathrm{trans}+T\right)}p_{\left(t\right)}{\mathrm{pent}}_{\left(T\right)}N_{\left(\cdot \right)}$	13	908.06	0.00	0.51	881.43
2.$\Phi_{\left(\mathrm{trans}+T\right)}p_{\left(t\right)}{\mathrm{pent}}_{\left(\cdot \right)}N_{\left(\cdot \right)}$	12	910.52	2.47	0.15	885.99
3.$\Phi_{\left(\mathrm{trans}\right)}p_{\left(t\right)}{\mathrm{pent}}_{\left(T\right)}N_{\left(\cdot \right)}$	12	910.94	2.88	0.12	886.41
4.$\Phi_{\left(\mathrm{trans}:T\right)}p_{\left(t\right)}{\mathrm{pent}}_{\left(T\right)}N_{\left(\cdot \right)}$	12	911.44	3.38	0.09	886.91
5.$\Phi_{\left(\mathrm{trans}+T\right)}p_{\left(t\right)}{\mathrm{pent}}_{\left(t\right)}N_{\left(\cdot \right)}$	17	912.75	4.69	0.05	877.70
6.$\Phi_{\left(\mathrm{trans}:T\right)}p_{\left(t\right)}{\mathrm{pent}}_{\left(\cdot \right)}N_{\left(\cdot \right)}$	11	913.65	5.60	0.03	891.20
7.$\Phi_{\left(\mathrm{trans}\right)}p_{\left(t\right)}{\mathrm{pent}}_{\left(\cdot \right)}N_{\left(\cdot \right)}$	11	913.89	5.83	0.03	891.44
8.$\Phi_{\left(\mathrm{trans}:T\right)}p_{\left(t\right)}{\mathrm{pent}}_{\left(t\right)}N_{\left(\cdot \right)}$	16	915.95	7.89	0.01	883.01
9.$\Phi_{\left(\mathrm{trans}\right)}p_{\left(t\right)}{\mathrm{pent}}_{\left(t\right)}N_{\left(\cdot \right)}$	16	915.95	7.89	0.01	883.01
10.$\Phi_{\left(\cdot \right)}p_{\left(t\right)}{\mathrm{pent}}_{\left(T\right)}N_{\left(.\right)}$	11	920.22	12.16	0.00	−550.11
11.$\Phi_{\left(T\right)}p_{\left(t\right)}{\mathrm{pent}}_{\left(T\right)}N_{\left(\cdot \right)}$	12	921.60	13.54	0.00	−550.81
12.$\Phi_{\left(\cdot \right)}p_{\left(t\right)}{\mathrm{pent}}_{\left(\cdot \right)}N_{\left(\cdot \right)}$	10	923.06	15.00	0.00	−545.20

Note

Models are ordered based on the Quasi‐likelihood AIC_C_ (QAIC_C_). *pent*: probability of recruitment; *trans*: transient effect. See Table 2 for other abbreviations. Full model list in Supporting Information Table A4.

## 4 DISCUSSION

Robust estimates of abundance and survival rates provide valuable information on population status, but they remain difficult to obtain for wide‐ranging, mobile species. This study stems from a photo‐identification database for fin whales in the JCP spanning 35 years. A mark–recapture analysis revealed a decline in apparent survival rates from 1990 to 2015. The deterioration in the condition of the population implied by this finding was supported by three additional observations: (a) a negative trend in recruitment from 2010 to 2016, (b) a significant decline in the super‐population size from an estimated 335 (95% CI 321–348) animals in 2004 to 2010 to 291 (95% CI 270–312) animals in 2010 to 2016, and (c) a sharp drop in the number of reported calves since 2008 with almost none since 2013. Before discussing possible explanations and implications of these findings, we first assess to what extent individual heterogeneity in site fidelity and movement patterns (e.g., nonrandom temporary emigration) could have influenced these results.

Open population models, such as the CJS and POPAN models used in this study, make two assumptions that require close scrutiny: (a) every marked animal present in the population at time (*t*) has the same probability of recapture (*p_t_*) and (b) all marked individuals have the same survival probability from one sampling occasion to the next (Hammond, 1990a; Lebreton et al., 1992). These assumptions are often unrealistic in wildlife studies due to inherent individual heterogeneity (Hammond, 1990a), but here several measures were applied to relax them. We accounted for trap‐dependency in the CJS recapture models and for a transient effect in the POPAN survival models to minimize potential bias. A pattern of trap‐dependence can appear in the data when parts of the study area are more frequently surveyed when animals have been detected, when individuals have higher recapture probabilities because they spend more time in more accessible parts of the study area, or when recapture probabilities are affected by life history (Pradel & Sanz‐Aguilar, 2012). Transient individuals were less likely to be biopsied, which is probably why no transient effect was found when limiting the dataset to biopsied individuals only. Compared to Ramp et al.'s (2014) estimate (*N* = 328; 95% CI 306–350), the inclusion of a transient effect did not alter the estimate of the super‐population size from 2004 to 2010 (*N* = 335; 95% CI 321–348), but was slightly more precise.

Despite accounting for trap‐dependency, there were strong indications of deviations from equal recapture probabilities in the first CJS model due to heterogeneity in the degree of site fidelity. Schaub et al. (2004) showed that while the U‐CARE Test2.CT component was initially developed to detect immediate trap response behavior; a significant test could also be indicative of Markovian temporary emigration. The inclusion of the time‐varying trap‐dependence variable (*m*) only approximately adjusts for temporary emigration because recapture probabilities are made dependent on sightings during the previous capture occasion (Schaub et al., 2004). However, Markovian temporary emigration is likely to be a lot more complex and may depend on other factors, such as prey availability. The AHC analysis revealed a range of site fidelity patterns, with two broad categories for core regulars and occasional visitors. As expected, the core regulars displayed higher probabilities of recapture compared to the occasional visitors. Hence, ignoring divergent site fidelity patterns equates to ignoring capture heterogeneity. Considering that apparent survival probabilities are a combination of true survival and permanent emigration, which in turn may be linked to site fidelity, estimates of survival rates may be biased when differences in site fidelity (and therefore recapture probability) are ignored. In particular, temporal variation in site fidelity could lead to misleading trends in apparent survival probabilities.

Another side effect of Markovian temporary emigration patterns is terminal bias in apparent survival estimates. Truncation of the time series of data, as suggested by Langtimm (2009), continued to show lower survival estimates at the end of the truncated time series, indicative of terminal bias. True survival and temporary emigration rates have both been shown to influence how far back in time the bias will propagate, where animals with high true survival and temporary emigration rates are most strongly affected by terminal bias (Langtimm, 2009). The negative trends in estimated survival were still evident when individuals were grouped into separate categories based on their site fidelity patterns, but occasional visitors showed a steady long‐term decline, while the survival of core regulars only dropped sharply in the final years of the study. It remains difficult to judge to what extent this drop was a result of remaining terminal bias. Models with a robust design (RD) can account for random temporary emigration and mitigate some of the concomitant bias (Kendall et al., 1997; Peñaloza et al., 2014). The RD requires individuals to be sampled at primary occasions, open to gains and losses, and closed secondary occasions. While the assumption of closure can be relaxed under specific conditions, Kendall (1999) found that estimators were biased when movement was nonrandom. In our case, preliminary analyses rejected the assumption of closure during shorter sampling periods (months) over summer (results not shown). The reason for this is considerable intra‐seasonal movement in and out of the study area. Due to the violation of the assumption of closure and the suspected presence of nonrandom temporary emigration, the RD model was considered inappropriate for this dataset. The Barker model (Barker, 1997) requires information on sightings between primary sampling periods, which was not available at the time of writing, but collaborations with other research institutions could make such an analysis possible in the future.

Such collaborative work would be particularly beneficial in the light of what we have learned from the results of this study. The increasing discovery curve (Figure 3) suggests that the JCP contains only a fraction of the animals in the GSL population. The limited data on photo‐ID matches among different study areas in the GSL highlight a high rate of interchange of individuals among areas. The high proportion of occasional visitors could be an indication that individuals are attracted to the JCP at times of high productivity. Coakes et al. (2005) described the occurrence of unusually high numbers of fin whales off Nova Scotia in 1997, including individuals from the GSL and Gulf of Maine, which coincided with higher levels of herring, sand lance, and euphausiid abundance. Concurrently, these authors reported a lower abundance of fin whales in the GSL in 1997. Estimates for the JCP have previously been considered representative for the GSL (Ramp et al., 2014); however, these results indicate that only about a quarter of the individuals (27.7%) show high site fidelity to the area, with the majority of fin whales using the JCP on an irregular basis, possibly depending on prey availability.

The problems encountered in the mark–recapture analysis are not unique to this study. We therefore caution researchers to consider possible effects of capture heterogeneity, introduced by divergent site fidelity patterns in their study population, and the effect of terminal bias when analyzing their data and interpreting the results of long‐term survival trends of long‐lived, highly mobile species. When alternative models (RD or Barker) are not applicable, the proposed categorization of individuals using cluster analysis offers a flexible alternative to minimize bias while providing additional insights into population demography. The grouping based on AHC analysis may also offer an alternative to “time since marking” (TSM) or 2 age‐class models. TSM models have been proposed to account for transients (animals seen only once) by estimating different survival rates for the first sampling interval compared to subsequent intervals (Pradel et al., 1997). However, site fidelity patterns can range along a continuum and by only accounting for transients, capture heterogeneity caused by individuals with low site fidelity may stay unaccounted for. In contrast, the AHC approach offers greater flexibility in defining groups based on their visitation patterns.

The median unisex estimate of survivorship of 0.946 (95% CI 0.910–0.967) was comparable to the estimate provided by Ramp *et al*. of 0.955 (95% CI 0.936–0.969). While the drop in apparent survival estimates in the later years of the study is likely subject to some terminal bias, the inference of a deterioration in population status is supported by the negative trend in recruitment and the significant decline in super‐population size, implying that a lower number of fin whales used the JCP at any point between 2010 and 2016 compared to 2004 and 2010. The decline in survival could partly be the result of higher rates of permanent emigration compared to immigration rates.

In the period of 2004 to 2010, 20 dead fin whales were reported in the GSL, compared to six animals for 2010 to 2016 (Quebec Marine Mammal Emergency Network Call Center, pers. comm.). These numbers are underestimates of true mortality rates because an unknown number of carcasses were missed or unreported. A documented cause of fin whale mortality is vessel collisions. Fin whales are the most commonly reported species in the current global vessel‐strike data set maintained by the Scientific Committee of the International Whaling Commission (Douglas et al., 2008; Laist, Knowlton, Mead, Collet, & Podesta, 2001; Panigada et al., 2006; Van Der Hoop et al., 2013). Such high mortality rates are likely unsustainable for a population of fewer than 300 individuals and could, in combination with the lack of recruitment, explain the observed decline in the super‐population size.

An alternative explanation is that the observed changes could be caused by varying environmental conditions affecting prey availability. Individuals may have permanently emigrated from the JCP in favor of a different feeding ground. A gradual shift in distribution could explain lower recruitment and a decrease in the super‐population size. While significant changes in the ecosystem of the GSL have been reported (Bui et al., 2010; Johnston et al., 2012; Ramp et al., 2015), it is not clear how fin whale prey availability changed over the study period or whether changes in prey distribution could have caused a shift in distribution away from the JCP in recent years. A shift in distribution would result in a concomitant increase in abundance in neighboring areas, which has not been confirmed (GREMM, pers. comm.), but large areas remain unsurveyed.

The new information presented here is important to consider in future conservation plans for fin whales in the GSL. Priority should be given to determining the underlying cause(s) of the observed decline, which we hypothesize to be the result of anthropogenic mortality events (e.g., vessel collisions, entanglements), changes in prey availability, or a combination of these factors. Failure to address these issues could result in an ongoing decline of fin whales in the GSL with unknown impacts on the balance of the GSL ecosystem.

## AUTHORS’ CONTRIBUTIONS

AS, CR, RS, PSH conceived the initial ideas of the study and designed the methodology; AC, CR, JD, MB, PJP, and RS collected photo‐identification data and/or skin samples; MB and PJP conducted the analysis to sex the individuals; JD, AC, CR, RS processed the photo‐ID data; AS analyzed the data; AS and PSH led the writing of the manuscript. All authors contributed critically to the drafts and gave final approval for publication.

## Data Availability

R code, capture histories, and sex of individual fin whales to estimate survival rates and abundance are available from the Dryad Digital Repository: https://doi.org/10.5061/dryad.pv93760.
