# Supplementary material for: Decline in abundance and apparent survival rates of fin whales (Balaenoptera physalus) in the northern Gulf of St. Lawrence
Source: Ecol Evol. 2019 Mar 15;9(7):4231–44. doi: 10.1002/ece3.5055 (PMC6468087; doi:10.1002/ece3.5055)
Supplement: Supplementary file 1 [file ECE3-9-4231-s001.docx]

**APPENDIX**

**Decline in abundance and apparent survival rates of fin whales (*Balaenoptera physalus*) in the northern Gulf of St. Lawrence**

Anna Schleimer^1,2,3*^, Christian Ramp^1,3^, Julien Delarue^3^, Alain Carpentier^3^, Martine Bérubé^2,4^, Per J. Palsbøll^2,4^, Richard Sears^3^, Philip S. Hammond^1^

^1^ Sea Mammal Research Unit, Scottish Oceans Institute, University of St Andrews, Fife, KY16 8LB UK

^2^ Marine Evolution and Conservation, Groningen Institute for Evolutionary Life Sciences, University of Groningen, Groningen, 9700 CC, The Netherlands

^3^ Mingan Island Cetacean Study, St Lambert, Québec, J4P 1T3, Canada

^4^ Center for Coastal Studies, 5 Holway Avenue, Provincetown, MA 02657, USA

***Corresponding author**: Anna Schleimer, [achs@st-andrews.ac.uk](mailto:achs@st-andrews.ac.uk)

Cormack-Jolly-Seber (CJS) models were fitted separately to individuals classified as core regulars or occasional visitors by the agglomerative hierarchical clustering analysis. After accounting for trap-dependence in the models fitted to the capture histories of core regular individuals (U-CARE Test2.CT, χ^2^ = 75.10, df = 24, p < 0.001), the over-dispersion factor was estimated to be $\hat{c}$ = 1.24 (overall χ^2^ = 70.7, df = 57). The model selection criterion (QAIC_C_) and standard errors were adjusted accordingly (Table A1). No $\hat{c}$ adjustment was necessary for the models fitted to the occasional visitor group ( χ^2^ = 87.22, df = 82, $\hat{c}$ = 1.06; Table A2). In both groups, the model for survival with a linear temporal trend ($\Phi_{(T)}$) and recapture probabilities accounting for time and trap dependence ($p_{(t+m)}$) held most of the weight.

**Table A1.** Model selection for CJS models fitted to capture histories from 1990 to 2016 for group of core regular individuals. See Table 2 for abbreviations.

| model | npar | QAICc | ΔQAICc | weight | QDeviance |
| --- | --- | --- | --- | --- | --- |
| 1. $\boldsymbol{\Phi}_{\boldsymbol{(}\boldsymbol{T}\boldsymbol{)}}\boldsymbol{p}_{\boldsymbol{(}\boldsymbol{t}\boldsymbol{+}\boldsymbol{m}\boldsymbol{)}}$ | 29 | 1857.19 | 0.00 | 0.97 | 1797.20 |
| 2. $\boldsymbol{\Phi}_{\boldsymbol{(.)}}\boldsymbol{p}_{\boldsymbol{(}\boldsymbol{t}\boldsymbol{+}\boldsymbol{m}\boldsymbol{)}}$ | 28 | 1863.86 | 6.67 | 0.03 | 1806.00 |
| 3. $\boldsymbol{\Phi}_{\boldsymbol{(}\boldsymbol{T}\boldsymbol{)}}\boldsymbol{p}_{\boldsymbol{(}\boldsymbol{t}\boldsymbol{)}}$ | 28 | 1876.04 | 18.85 | 0.00 | 1423.55 |
| 4. $\boldsymbol{\Phi}_{\boldsymbol{(.)}}\boldsymbol{p}_{\boldsymbol{(}\boldsymbol{t}\boldsymbol{)}}$ | 27 | 1892.16 | 34.97 | 0.00 | 1441.79 |
| 5. $\boldsymbol{\Phi}_{\boldsymbol{(}\boldsymbol{t}\boldsymbol{)}}\boldsymbol{p}_{\boldsymbol{(}\boldsymbol{t}\boldsymbol{+}\boldsymbol{m}\boldsymbol{)}}$ | 53 | 1904.65 | 47.46 | 0.00 | 1791.90 |
| 6. $\boldsymbol{\Phi}_{\boldsymbol{(}\boldsymbol{t}\boldsymbol{)}}\boldsymbol{p}_{\boldsymbol{(}\boldsymbol{t}\boldsymbol{)}}$ | 52 | 1923.68 | 66.49 | 0.00 | 1418.56 |
| 7. $\boldsymbol{\Phi}_{\boldsymbol{(}\boldsymbol{T}\boldsymbol{)}}\boldsymbol{p}_{\boldsymbol{(}\boldsymbol{m}\boldsymbol{)}}$ | 4 | 1966.51 | 109.32 | 0.00 | 1958.47 |
| 8. $\boldsymbol{\Phi}_{\boldsymbol{(.)}}\boldsymbol{p}_{\boldsymbol{(}\boldsymbol{m}\boldsymbol{)}}$ | 3 | 1967.65 | 110.47 | 0.00 | 1961.63 |
| 9. $\boldsymbol{\Phi}_{\boldsymbol{(}\boldsymbol{t}\boldsymbol{)}}\boldsymbol{p}_{\boldsymbol{(}\boldsymbol{m}\boldsymbol{)}}$ | 28 | 2011.31 | 154.12 | 0.00 | 1953.45 |

**Table A2.** Model selection for CJS models fitted to capture histories from 1990 to 2016 for group of occasional visitors. See Table 2 for abbreviations.

| model | npar | AICc | ΔAICc | weight | Deviance |
| --- | --- | --- | --- | --- | --- |
| 1. $\boldsymbol{\Phi}_{\boldsymbol{(}\boldsymbol{T}\boldsymbol{)}}\boldsymbol{p}_{\boldsymbol{(}\boldsymbol{t}\boldsymbol{+}\boldsymbol{m}\boldsymbol{)}}$ | 29 | 2814.53 | 0.00 | 0.77 | 2754.20 |
| 2. $\boldsymbol{\Phi}_{\boldsymbol{(.)}}\boldsymbol{p}_{\boldsymbol{(}\boldsymbol{t}\boldsymbol{+}\boldsymbol{m}\boldsymbol{)}}$ | 28 | 2817.43 | 2.89 | 0.18 | 2759.26 |
| 3. $\boldsymbol{\Phi}_{\boldsymbol{(}\boldsymbol{T}\boldsymbol{)}}\boldsymbol{p}_{\boldsymbol{(}\boldsymbol{t}\boldsymbol{)}}$ | 28 | 2820.41 | 5.88 | 0.04 | 1517.39 |
| 4. $\boldsymbol{\Phi}_{\boldsymbol{(.)}}\boldsymbol{p}_{\boldsymbol{(}\boldsymbol{t}\boldsymbol{)}}$ | 27 | 2826.34 | 11.81 | 0.00 | 1525.47 |
| 5. $\boldsymbol{\Phi}_{\boldsymbol{(}\boldsymbol{t}\boldsymbol{)}}\boldsymbol{p}_{\boldsymbol{(}\boldsymbol{t}\boldsymbol{+}\boldsymbol{m}\boldsymbol{)}}$ | 53 | 2849.85 | 35.32 | 0.00 | 2735.94 |
| 6. $\boldsymbol{\Phi}_{\boldsymbol{(}\boldsymbol{t}\boldsymbol{)}}\boldsymbol{p}_{\boldsymbol{(}\boldsymbol{t}\boldsymbol{)}}$ | 52 | 2854.29 | 39.76 | 0.00 | 1497.83 |
| 7. $\boldsymbol{\Phi}_{\boldsymbol{(.)}}\boldsymbol{p}_{\boldsymbol{(}\boldsymbol{m}\boldsymbol{)}}$ | 3 | 2997.16 | 182.63 | 0.00 | 2991.13 |
| 8. $\boldsymbol{\Phi}_{\boldsymbol{(}\boldsymbol{T}\boldsymbol{)}}\boldsymbol{p}_{\boldsymbol{(}\boldsymbol{m}\boldsymbol{)}}$ | 4 | 2998.38 | 183.84 | 0.00 | 2990.32 |
| 9. $\boldsymbol{\Phi}_{\boldsymbol{(}\boldsymbol{t}\boldsymbol{)}}\boldsymbol{p}_{\boldsymbol{(}\boldsymbol{m}\boldsymbol{)}}$ | 28 | 3017.75 | 203.22 | 0.00 | 2959.58 |

¨

To get comparable estimates to the period of 2010 to 2016, a POPAN model with transient effect was fitted to data from 2004 to 2010 to estimate the super-population size $N$. A CJS model was fitted for approximate GOF testing in U-CARE. After accounting for a transient effect (U-CARE Test3.SR, χ^2^ = 16.04, df = 5, p = 0.007), there was no sign of over-dispersion left in the data (χ^2^ = 14.87, df = 14, $\hat{c}$ = 1.06) and no $\hat{c}$ adjustment was applied (Table S3).

**Table A3.** Model selection for POPAN 2004 to 2010.

| model | npar | AICc | ΔAICc | weight | Deviance |
| --- | --- | --- | --- | --- | --- |
| 1. $\boldsymbol{\Phi}_{\boldsymbol{(}\boldsymbol{trans}\boldsymbol{:}\boldsymbol{T}\boldsymbol{)}}\boldsymbol{p}_{\boldsymbol{(}\boldsymbol{t}\boldsymbol{)}}\boldsymbol{pent}_{\boldsymbol{(}\boldsymbol{t}\boldsymbol{)}}\boldsymbol{N}_{\boldsymbol{(.)}}$ | 16 | 1726.64 | 0.00 | 0.72 | 1693.97 |
| 2. $\boldsymbol{\Phi}_{\boldsymbol{(}\boldsymbol{trans}\boldsymbol{+}\boldsymbol{T}\boldsymbol{)}}\boldsymbol{p}_{\boldsymbol{(}\boldsymbol{t}\boldsymbol{)}}\boldsymbol{pent}_{\boldsymbol{(}\boldsymbol{t}\boldsymbol{)}}\boldsymbol{N}_{\boldsymbol{(.)}}$ | 17 | 1729.64 | 3.00 | 0.16 | 1694.88 |
| 3. $\boldsymbol{\Phi}_{\boldsymbol{(}\boldsymbol{trans}\boldsymbol{+}\boldsymbol{T}\boldsymbol{)}}\boldsymbol{p}_{\boldsymbol{(.)}}\boldsymbol{pent}_{\boldsymbol{(}\boldsymbol{t}\boldsymbol{)}}\boldsymbol{N}_{\boldsymbol{(.)}}$ | 11 | 1730.36 | 3.71 | 0.11 | 1708.03 |
| 4. $\boldsymbol{\Phi}_{\boldsymbol{(}\boldsymbol{trans}\boldsymbol{:}\boldsymbol{T}\boldsymbol{)}}\boldsymbol{p}_{\boldsymbol{(}\boldsymbol{t}\boldsymbol{)}}\boldsymbol{pent}_{\boldsymbol{(}\boldsymbol{T}\boldsymbol{)}}\boldsymbol{N}_{\boldsymbol{(.)}}$ | 12 | 1741.31 | 14.67 | 0.00 | 1716.93 |
| 5. $\boldsymbol{\Phi}_{\boldsymbol{(}\boldsymbol{trans}\boldsymbol{:}\boldsymbol{T}\boldsymbol{)}}\boldsymbol{p}_{\boldsymbol{(.)}}\boldsymbol{pent}_{\boldsymbol{(}\boldsymbol{t}\boldsymbol{)}}\boldsymbol{N}_{\boldsymbol{(.)}}$ | 10 | 1742.08 | 15.44 | 0.00 | 1721.81 |
| 6. $\boldsymbol{\Phi}_{\boldsymbol{(}\boldsymbol{T}\boldsymbol{)}}\boldsymbol{p}_{\boldsymbol{(.)}}\boldsymbol{pent}_{\boldsymbol{(}\boldsymbol{t}\boldsymbol{)}}\boldsymbol{N}_{\boldsymbol{(.)}}$ | 10 | 1744.44 | 17.79 | 0.00 | -787.38 |
| 7. $\boldsymbol{\Phi}_{\boldsymbol{(}\boldsymbol{trans}\boldsymbol{+}\boldsymbol{T}\boldsymbol{)}}\boldsymbol{p}_{\boldsymbol{(}\boldsymbol{t}\boldsymbol{)}}\boldsymbol{pent}_{\boldsymbol{(}\boldsymbol{T}\boldsymbol{)}}\boldsymbol{N}_{\boldsymbol{(.)}}$ | 13 | 1744.63 | 17.99 | 0.00 | 1718.19 |
| 8. $\boldsymbol{\Phi}_{\boldsymbol{(}\boldsymbol{T}\boldsymbol{)}}\boldsymbol{p}_{\boldsymbol{(}\boldsymbol{t}\boldsymbol{)}}\boldsymbol{pent}_{\boldsymbol{(}\boldsymbol{t}\boldsymbol{)}}\boldsymbol{N}_{\boldsymbol{(.)}}$ | 16 | 1744.77 | 18.12 | 0.00 | -799.46 |
| 9. $\boldsymbol{\Phi}_{\boldsymbol{(}\boldsymbol{trans}\boldsymbol{)}}\boldsymbol{p}_{\boldsymbol{(}\boldsymbol{t}\boldsymbol{)}}\boldsymbol{pent}_{\boldsymbol{(}\boldsymbol{t}\boldsymbol{)}}\boldsymbol{N}_{\boldsymbol{(.)}}$ | 16 | 1747.66 | 21.02 | 0.00 | 1714.99 |
| 10. $\boldsymbol{\Phi}_{\boldsymbol{(}\boldsymbol{.}\boldsymbol{)}}\boldsymbol{p}_{\boldsymbol{(}\boldsymbol{t}\boldsymbol{)}}\boldsymbol{pent}_{\boldsymbol{(}\boldsymbol{t}\boldsymbol{)}}\boldsymbol{N}_{\boldsymbol{(.)}}$ | 15 | 1749.73 | 23.08 | 0.00 | -792.42 |
| 11. $\boldsymbol{\Phi}_{\boldsymbol{(}\boldsymbol{T}\boldsymbol{)}}\boldsymbol{p}_{\boldsymbol{(}\boldsymbol{t}\boldsymbol{)}}\boldsymbol{pent}_{\boldsymbol{(}\boldsymbol{T}\boldsymbol{)}}\boldsymbol{N}_{\boldsymbol{(.)}}$ | 12 | 1758.99 | 32.34 | 0.00 | -776.95 |
| 12. $\boldsymbol{\Phi}_{\boldsymbol{(}\boldsymbol{trans}\boldsymbol{)}}\boldsymbol{p}_{\boldsymbol{(}\boldsymbol{t}\boldsymbol{)}}\boldsymbol{pent}_{\boldsymbol{(}\boldsymbol{T}\boldsymbol{)}}\boldsymbol{N}_{\boldsymbol{(.)}}$ | 12 | 1762.61 | 35.97 | 0.00 | 1738.23 |
| 13. $\boldsymbol{\Phi}_{\boldsymbol{(}\boldsymbol{trans}\boldsymbol{:}\boldsymbol{T}\boldsymbol{)}}\boldsymbol{p}_{\boldsymbol{(}\boldsymbol{t}\boldsymbol{)}}\boldsymbol{pent}_{\boldsymbol{(.)}}\boldsymbol{N}_{\boldsymbol{(.)}}$ | 11 | 1763.74 | 37.10 | 0.00 | 1741.42 |
| 14. $\boldsymbol{\Phi}_{\boldsymbol{(.)}}\boldsymbol{p}_{\boldsymbol{(}\boldsymbol{t}\boldsymbol{)}}\boldsymbol{pent}_{\boldsymbol{(}\boldsymbol{T}\boldsymbol{)}}\boldsymbol{N}_{\boldsymbol{(.)}}$ | 11 | 1764.38 | 37.74 | 0.00 | -769.49 |
| 15. $\boldsymbol{\Phi}_{\boldsymbol{(}\boldsymbol{trans}\boldsymbol{+}\boldsymbol{T}\boldsymbol{)}}\boldsymbol{p}_{\boldsymbol{(.)}}\boldsymbol{pent}_{\boldsymbol{(}\boldsymbol{T}\boldsymbol{)}}\boldsymbol{N}_{\boldsymbol{(.)}}$ | 7 | 1764.96 | 38.31 | 0.00 | 1750.82 |
| 16. $\boldsymbol{\Phi}_{\boldsymbol{(}\boldsymbol{trans}\boldsymbol{+}\boldsymbol{T}\boldsymbol{)}}\boldsymbol{p}_{\boldsymbol{(}\boldsymbol{t}\boldsymbol{)}}\boldsymbol{pent}_{\boldsymbol{(.)}}\boldsymbol{N}_{\boldsymbol{(.)}}$ | 12 | 1767.20 | 40.55 | 0.00 | 1742.81 |
| 17. $\boldsymbol{\Phi}_{\boldsymbol{(.)}}\boldsymbol{p}_{\boldsymbol{(.)}}\boldsymbol{pent}_{\boldsymbol{(}\boldsymbol{t}\boldsymbol{)}}\boldsymbol{N}_{\boldsymbol{(.)}}$ | 9 | 1767.63 | 40.98 | 0.00 | -762.14 |
| 18. $\boldsymbol{\Phi}_{\boldsymbol{(}\boldsymbol{trans}\boldsymbol{)}}\boldsymbol{p}_{\boldsymbol{(.)}}\boldsymbol{pent}_{\boldsymbol{(}\boldsymbol{t}\boldsymbol{)}}\boldsymbol{N}_{\boldsymbol{(.)}}$ | 10 | 1769.33 | 42.69 | 0.00 | 1749.06 |
| 19. $\boldsymbol{\Phi}_{\boldsymbol{(}\boldsymbol{trans}\boldsymbol{:}\boldsymbol{T}\boldsymbol{)}}\boldsymbol{p}_{\boldsymbol{(.)}}\boldsymbol{pent}_{\boldsymbol{(}\boldsymbol{T}\boldsymbol{)}}\boldsymbol{N}_{\boldsymbol{(.)}}$ | 6 | 1777.94 | 51.29 | 0.00 | 1765.83 |
| 20. $\boldsymbol{\Phi}_{\boldsymbol{(}\boldsymbol{T}\boldsymbol{)}}\boldsymbol{p}_{\boldsymbol{(.)}}\boldsymbol{pent}_{\boldsymbol{(}\boldsymbol{T}\boldsymbol{)}}\boldsymbol{N}_{\boldsymbol{(.)}}$ | 6 | 1779.08 | 52.43 | 0.00 | -744.58 |
| 21. $\boldsymbol{\Phi}_{\boldsymbol{(}\boldsymbol{T}\boldsymbol{)}}\boldsymbol{p}_{\boldsymbol{(}\boldsymbol{t}\boldsymbol{)}}\boldsymbol{pent}_{\boldsymbol{(}\boldsymbol{.}\boldsymbol{)}}\boldsymbol{N}_{\boldsymbol{(.)}}$ | 11 | 1782.38 | 55.74 | 0.00 | -751.49 |
| 22. $\boldsymbol{\Phi}_{\boldsymbol{(}\boldsymbol{trans}\boldsymbol{)}}\boldsymbol{p}_{\boldsymbol{(}\boldsymbol{t}\boldsymbol{)}}\boldsymbol{pent}_{\boldsymbol{(.)}}\boldsymbol{N}_{\boldsymbol{(.)}}$ | 11 | 1785.75 | 59.11 | 0.00 | 1763.43 |
| 23. $\boldsymbol{\Phi}_{\boldsymbol{(.)}}\boldsymbol{p}_{\boldsymbol{(}\boldsymbol{t}\boldsymbol{)}}\boldsymbol{pent}_{\boldsymbol{(.)}}\boldsymbol{N}_{\boldsymbol{(.)}}$ | 10 | 1788.31 | 61.67 | 0.00 | -743.51 |
| 24. $\boldsymbol{\Phi}_{\boldsymbol{(}\boldsymbol{trans}\boldsymbol{+}\boldsymbol{T}\boldsymbol{)}}\boldsymbol{p}_{\boldsymbol{(.)}}\boldsymbol{pent}_{\boldsymbol{(.)}}\boldsymbol{N}_{\boldsymbol{(.)}}$ | 6 | 1797.69 | 71.04 | 0.00 | 1785.58 |
| 25. $\boldsymbol{\Phi}_{\boldsymbol{(.)}}\boldsymbol{p}_{\boldsymbol{(.)}}\boldsymbol{pent}_{\boldsymbol{(}\boldsymbol{T}\boldsymbol{)}}\boldsymbol{N}_{\boldsymbol{(.)}}$ | 5 | 1803.50 | 76.86 | 0.00 | -718.12 |
| 26. $\boldsymbol{\Phi}_{\boldsymbol{(}\boldsymbol{trans}\boldsymbol{)}}\boldsymbol{p}_{\boldsymbol{(.)}}\boldsymbol{pent}_{\boldsymbol{(}\boldsymbol{T}\boldsymbol{)}}\boldsymbol{N}_{\boldsymbol{(.)}}$ | 6 | 1805.16 | 78.52 | 0.00 | 1793.06 |
| 27. $\boldsymbol{\Phi}_{\boldsymbol{(}\boldsymbol{T}\boldsymbol{)}}\boldsymbol{p}_{\boldsymbol{(.)}}\boldsymbol{pent}_{\boldsymbol{(}\boldsymbol{.}\boldsymbol{)}}\boldsymbol{N}_{\boldsymbol{(.)}}$ | 5 | 1811.31 | 84.67 | 0.00 | -710.31 |
| 28. $\boldsymbol{\Phi}_{\boldsymbol{(}\boldsymbol{trans}\boldsymbol{:}\boldsymbol{T}\boldsymbol{)}}\boldsymbol{p}_{\boldsymbol{(.)}}\boldsymbol{pent}_{\boldsymbol{(}\boldsymbol{t}\boldsymbol{)}}\boldsymbol{N}_{\boldsymbol{(.)}}$ | 5 | 1818.83 | 92.19 | 0.00 | 1808.76 |
| 29. $\boldsymbol{\Phi}_{\boldsymbol{(.)}}\boldsymbol{p}_{\boldsymbol{(.)}}\boldsymbol{pent}_{\boldsymbol{(.)}}\boldsymbol{N}_{\boldsymbol{(.)}}$ | 4 | 1844.10 | 117.45 | 0.00 | -675.51 |
| 30. $\boldsymbol{\Phi}_{\boldsymbol{(}\boldsymbol{trans}\boldsymbol{)}}\boldsymbol{p}_{\boldsymbol{(.)}}\boldsymbol{pent}_{\boldsymbol{(.)}}\boldsymbol{N}_{\boldsymbol{(.)}}$ | 5 | 1845.29 | 118.64 | 0.00 | 1835.21 |

**Table A4.** Full list of POPAN models considered for recapture histories from 2010 to 2016.

| model | npar | QAICc | ΔQAICc | weight | QDeviance |
| --- | --- | --- | --- | --- | --- |
| 1. $\boldsymbol{\Phi}_{\mathbf{(}\boldsymbol{trans}\mathbf{+}\boldsymbol{T}\mathbf{)}}\boldsymbol{p}_{\mathbf{(}\boldsymbol{t}\mathbf{)}}\boldsymbol{pent}_{\mathbf{(}\boldsymbol{T}\mathbf{)}}\boldsymbol{N}_{\mathbf{(.)}}$ | 13 | 908.06 | 0.00 | 0.51 | 881.43 |
| 2. $\boldsymbol{\Phi}_{\mathbf{(}\boldsymbol{trans}\mathbf{+}\boldsymbol{T}\mathbf{)}}\boldsymbol{p}_{\mathbf{(}\boldsymbol{t}\mathbf{)}}\boldsymbol{pent}_{\mathbf{(.)}}\boldsymbol{N}_{\mathbf{(.)}}$ | 12 | 910.52 | 2.47 | 0.15 | 885.99 |
| 3. $\boldsymbol{\Phi}_{\mathbf{(}\boldsymbol{trans}\mathbf{)}}\boldsymbol{p}_{\mathbf{(}\boldsymbol{t}\mathbf{)}}\boldsymbol{pent}_{\mathbf{(}\boldsymbol{T}\mathbf{)}}\boldsymbol{N}_{\mathbf{(.)}}$ | 12 | 910.94 | 2.88 | 0.12 | 886.41 |
| 4. $\boldsymbol{\Phi}_{\mathbf{(}\boldsymbol{trans}\mathbf{:}\boldsymbol{T}\mathbf{)}}\boldsymbol{p}_{\mathbf{(}\boldsymbol{t}\mathbf{)}}\boldsymbol{pent}_{\mathbf{(}\boldsymbol{T}\mathbf{)}}\boldsymbol{N}_{\mathbf{(.)}}$ | 12 | 911.44 | 3.38 | 0.09 | 886.91 |
| 5. $\boldsymbol{\Phi}_{\mathbf{(}\boldsymbol{trans}\mathbf{+}\boldsymbol{T}\mathbf{)}}\boldsymbol{p}_{\mathbf{(}\boldsymbol{t}\mathbf{)}}\boldsymbol{pent}_{\mathbf{(}\boldsymbol{t}\mathbf{)}}\boldsymbol{N}_{\mathbf{(.)}}$ | 17 | 912.75 | 4.69 | 0.05 | 877.70 |
| 6. $\boldsymbol{\Phi}_{\mathbf{(}\boldsymbol{trans}\mathbf{:}\boldsymbol{T}\mathbf{)}}\boldsymbol{p}_{\mathbf{(}\boldsymbol{t}\mathbf{)}}\boldsymbol{pent}_{\mathbf{(.)}}\boldsymbol{N}_{\mathbf{(.)}}$ | 11 | 913.65 | 5.60 | 0.03 | 891.20 |
| 7. $\boldsymbol{\Phi}_{\mathbf{(}\boldsymbol{trans}\mathbf{)}}\boldsymbol{p}_{\mathbf{(}\boldsymbol{t}\mathbf{)}}\boldsymbol{pent}_{\mathbf{(.)}}\boldsymbol{N}_{\mathbf{(.)}}$ | 11 | 913.89 | 5.83 | 0.03 | 891.44 |
| 8. $\boldsymbol{\Phi}_{\mathbf{(}\boldsymbol{trans}\mathbf{:}\boldsymbol{T}\mathbf{)}}\boldsymbol{p}_{\mathbf{(}\boldsymbol{t}\mathbf{)}}\boldsymbol{pent}_{\mathbf{(}\boldsymbol{t}\mathbf{)}}\boldsymbol{N}_{\mathbf{(.)}}$ | 16 | 915.95 | 7.89 | 0.01 | 883.01 |
| 9. $\boldsymbol{\Phi}_{\mathbf{(}\boldsymbol{trans}\mathbf{)}}\boldsymbol{p}_{\mathbf{(}\boldsymbol{t}\mathbf{)}}\boldsymbol{pent}_{\mathbf{(}\boldsymbol{t}\mathbf{)}}\boldsymbol{N}_{\mathbf{(.)}}$ | 16 | 915.95 | 7.89 | 0.01 | 883.01 |
| 10. $\boldsymbol{\Phi}_{\mathbf{(.)}}\boldsymbol{p}_{\mathbf{(}\boldsymbol{t}\mathbf{)}}\boldsymbol{pent}_{\mathbf{(}\boldsymbol{T}\mathbf{)}}\boldsymbol{N}_{\mathbf{(.)}}$ | 11 | 920.22 | 12.16 | 0.00 | -550.11 |
| 11. $\boldsymbol{\Phi}_{\mathbf{(}\boldsymbol{T}\mathbf{)}}\boldsymbol{p}_{\mathbf{(}\boldsymbol{t}\mathbf{)}}\boldsymbol{pent}_{\mathbf{(}\boldsymbol{T}\mathbf{)}}\boldsymbol{N}_{\mathbf{(.)}}$ | 12 | 921.60 | 13.54 | 0.00 | -550.81 |
| 12. $\boldsymbol{\Phi}_{\mathbf{(}\mathbf{.}\mathbf{)}}\boldsymbol{p}_{\mathbf{(}\boldsymbol{t}\mathbf{)}}\boldsymbol{pent}_{\mathbf{(.)}}\boldsymbol{N}_{\mathbf{(.)}}$ | 10 | 923.06 | 15.00 | 0.00 | -545.20 |
| 13. $\boldsymbol{\Phi}_{\boldsymbol{(}\boldsymbol{T}\boldsymbol{)}}\boldsymbol{p}_{\boldsymbol{(}\boldsymbol{t}\boldsymbol{)}}\boldsymbol{pent}_{\boldsymbol{(}\boldsymbol{.}\boldsymbol{)}}\boldsymbol{N}_{\boldsymbol{(.)}}$ | 11 | 923.83 | 15.78 | 0.00 | -546.50 |
| 14. $\boldsymbol{\Phi}_{\boldsymbol{(.)}}\boldsymbol{p}_{\boldsymbol{(}\boldsymbol{t}\boldsymbol{)}}\boldsymbol{pent}_{\boldsymbol{(}\boldsymbol{t}\boldsymbol{)}}\boldsymbol{N}_{\boldsymbol{(.)}}$ | 15 | 925.02 | 16.96 | 0.00 | -553.69 |
| 15. $\boldsymbol{\Phi}_{\boldsymbol{(}\boldsymbol{T}\boldsymbol{)}}\boldsymbol{p}_{\boldsymbol{(}\boldsymbol{t}\boldsymbol{)}}\boldsymbol{pent}_{\boldsymbol{(}\boldsymbol{t}\boldsymbol{)}}\boldsymbol{N}_{\boldsymbol{(.)}}$ | 16 | 926.30 | 18.25 | 0.00 | -554.51 |
| 16. $\boldsymbol{\Phi}_{\boldsymbol{(}\boldsymbol{trans}\boldsymbol{+}\boldsymbol{T}\boldsymbol{)}}\boldsymbol{p}_{\boldsymbol{(.)}}\boldsymbol{pent}_{\boldsymbol{(.)}}\boldsymbol{N}_{\boldsymbol{(.)}}$ | 6 | 935.21 | 27.15 | 0.00 | 923.06 |
| 17. $\boldsymbol{\Phi}_{\boldsymbol{(}\boldsymbol{trans}\boldsymbol{)}}\boldsymbol{p}_{\boldsymbol{(.)}}\boldsymbol{pent}_{\boldsymbol{(.)}}\boldsymbol{N}_{\boldsymbol{(.)}}$ | 5 | 935.33 | 27.27 | 0.00 | 925.23 |
| 18. $\boldsymbol{\Phi}_{\boldsymbol{(}\boldsymbol{trans}\boldsymbol{)}}\boldsymbol{p}_{\boldsymbol{(.)}}\boldsymbol{pent}_{\boldsymbol{(}\boldsymbol{T}\boldsymbol{)}}\boldsymbol{N}_{\boldsymbol{(.)}}$ | 6 | 936.10 | 28.04 | 0.00 | 923.96 |
| 19. $\boldsymbol{\Phi}_{\mathbf{(}\boldsymbol{trans}\mathbf{+}\boldsymbol{T}\mathbf{)}}\boldsymbol{p}_{\mathbf{(}\boldsymbol{.}\mathbf{)}}\boldsymbol{pent}_{\mathbf{(}\boldsymbol{T}\mathbf{)}}\boldsymbol{N}_{\mathbf{(.)}}$ | 7 | 936.19 | 28.13 | 0.00 | 922.00 |
| 20. $\boldsymbol{\Phi}_{\boldsymbol{(}\boldsymbol{trans}\boldsymbol{)}}\boldsymbol{p}_{\boldsymbol{(.)}}\boldsymbol{pent}_{\boldsymbol{(}\boldsymbol{t}\boldsymbol{)}}\boldsymbol{N}_{\boldsymbol{(.)}}$ | 10 | 938.35 | 30.30 | 0.00 | 917.98 |
| 21. $\boldsymbol{\Phi}_{\mathbf{(}\boldsymbol{trans}\mathbf{+}\boldsymbol{T}\mathbf{)}}\boldsymbol{p}_{\mathbf{(}\boldsymbol{.}\mathbf{)}}\boldsymbol{pent}_{\mathbf{(}\boldsymbol{t}\mathbf{)}}$ | 11 | 938.48 | 30.42 | 0.00 | 916.03 |
| 22. $\boldsymbol{\Phi}_{\mathbf{(}\boldsymbol{trans}\mathbf{:}\boldsymbol{T}\mathbf{)}}\boldsymbol{p}_{\mathbf{(}\boldsymbol{.}\mathbf{)}}\boldsymbol{pent}_{\mathbf{(}\boldsymbol{T}\mathbf{)}}\boldsymbol{N}_{\mathbf{(.)}}$ | 6 | 940.22 | 32.16 | 0.00 | 928.07 |
| 23. $\boldsymbol{\Phi}_{\mathbf{(}\boldsymbol{trans}\mathbf{:}\boldsymbol{T}\mathbf{)}}\boldsymbol{p}_{\mathbf{(}\boldsymbol{.}\mathbf{)}}\boldsymbol{pent}_{\mathbf{(}\boldsymbol{.}\mathbf{)}}\boldsymbol{N}_{\mathbf{(.)}}$ | 5 | 940.24 | 32.18 | 0.00 | 930.13 |
| 24. $\boldsymbol{\Phi}_{\mathbf{(}\boldsymbol{trans}\mathbf{:}\boldsymbol{T}\mathbf{)}}\boldsymbol{p}_{\mathbf{(}\boldsymbol{.}\mathbf{)}}\boldsymbol{pent}_{\mathbf{(}\boldsymbol{t}\mathbf{)}}\boldsymbol{N}_{\mathbf{(.)}}$ | 10 | 942.40 | 34.34 | 0.00 | 922.02 |
| 25. $\boldsymbol{\Phi}_{\boldsymbol{(.)}}\boldsymbol{p}_{\boldsymbol{(.)}}\boldsymbol{pent}_{\boldsymbol{(}\boldsymbol{.}\boldsymbol{)}}\boldsymbol{N}_{\boldsymbol{(.)}}$ | 4 | 946.61 | 38.55 | 0.00 | -509.34 |
| 26. $\boldsymbol{\Phi}_{\boldsymbol{(.)}}\boldsymbol{p}_{\boldsymbol{(.)}}\boldsymbol{pent}_{\boldsymbol{(}\boldsymbol{T}\boldsymbol{)}}\boldsymbol{N}_{\boldsymbol{(.)}}$ | 5 | 946.99 | 38.93 | 0.00 | -510.99 |
| 27. $\boldsymbol{\Phi}_{\boldsymbol{(}\boldsymbol{T}\boldsymbol{)}}\boldsymbol{p}_{\boldsymbol{(.)}}\boldsymbol{pent}_{\boldsymbol{(.)}}\boldsymbol{N}_{\boldsymbol{(.)}}$ | 5 | 948.59 | 40.53 | 0.00 | -509.39 |
| 28. $\boldsymbol{\Phi}_{\boldsymbol{(}\boldsymbol{T}\boldsymbol{)}}\boldsymbol{p}_{\boldsymbol{(.)}}\boldsymbol{pent}_{\boldsymbol{(}\boldsymbol{T}\boldsymbol{)}}\boldsymbol{N}_{\boldsymbol{(.)}}$ | 6 | 948.70 | 40.64 | 0.00 | -511.33 |
| 29. $\boldsymbol{\Phi}_{\boldsymbol{(.)}}\boldsymbol{p}_{\boldsymbol{(.)}}\boldsymbol{pent}_{\boldsymbol{(}\boldsymbol{t}\boldsymbol{)}}\boldsymbol{N}_{\boldsymbol{(.)}}$ | 9 | 949.18 | 41.12 | 0.00 | -517.01 |
| 30. $\boldsymbol{\Phi}_{\boldsymbol{(}\boldsymbol{T}\boldsymbol{)}}\boldsymbol{p}_{\boldsymbol{(.)}}\boldsymbol{pent}_{\boldsymbol{(}\boldsymbol{t}\boldsymbol{)}}\boldsymbol{N}_{\boldsymbol{(.)}}$ | 10 | 950.86 | 42.80 | 0.00 | -517.40 |
